# Expression of the Antimicrobial Peptide Piscidin 1 and Neuropeptides in Fish Gill and Skin: A Potential Participation in Neuro-Immune Interaction

**DOI:** 10.3390/md20020145

**Published:** 2022-02-17

**Authors:** Giacomo Zaccone, Gioele Capillo, Jorge Manuel Oliveira Fernandes, Viswanath Kiron, Eugenia Rita Lauriano, Alessio Alesci, Patrizia Lo Cascio, Maria Cristina Guerrera, Michal Kuciel, Krystyna Zuwala, Jose Manuel Icardo, Atsushi Ishimatsu, Ryosuke Murata, Takafumi Amagai, Antonino Germanà, Marialuisa Aragona

**Affiliations:** 1 Department of Veterinary Sciences, University of Messina, Polo Universitario dell’Annunziata, 98168 Messina, Italy; gcapillo@unime.it (G.C.); mguerrera@unime.it (M.C.G.); agermana@unime.it (A.G.); mlaragona@unime.it (M.A.); 2 Institute for Marine Biological Resources and Biotechnology (IRBIM), National Research Council (CNR), 98122 Messina, Italy; 3 Faculty of Biosciences and Aquaculture, Nord University, 8049 Bodø, Norway; kiron.viswanath@nord.no; 4 Department of Chemical, Biological, Pharmaceutical and Environmental Sciences, University of Messina, 98168 Messina, Italy; elauriano@unime.it (E.R.L.); alessio.alesci@gmail.com (A.A.); plocascio@unime.it (P.L.C.); 5 Poison Information Center, Department of Toxicology and Environmental Disease, Faculty of Medicine, Jagellonian University, Kopernika 15, 30501 Cracow, Poland; michalkuciel@gmail.com; 6 Department of Comparative Anatomy, Faculty of Biology, Institute of Zoology and Biomedical Research, Jagellonian University, 30387 Cracow, Poland; krystyna.zuwala@uj.edu.pl; 7 Department of Anatomy and Cell Biology, Poligono de Cazona, Faculty of Medicine, University of Cantabria, 39011 Santander, Spain; 8 Graduate School of Fisheries and Environmental Sciences, Nagasaki University, 1-14 Bunkyo-machi, Nagasaki 852-8521, Japan; a-ishima@nagasaki-u.ac.jp; 9 Institute for East China Sea Research, Nagasaki University, Nagasaki 852-8521, Japan; murata-r@nagasaki-u.ac.jp (R.M.); tamagai@nagasaki-u.ac.jp (T.A.)

**Keywords:** Pis-IR/gene expression, neuropeptides, NECs, peripheral nervous system, mast cells, eosinophils, mucus, gill, skin, fish

## Abstract

Antimicrobial peptides (AMPs) are found widespread in nature and possess antimicrobial and immunomodulatory activities. Due to their multifunctional properties, these peptides are a focus of growing body of interest and have been characterized in several fish species. Due to their similarities in amino-acid composition and amphipathic design, it has been suggested that neuropeptides may be directly involved in the innate immune response against pathogen intruders. In this review, we report the molecular characterization of the fish-specific AMP piscidin1, the production of an antibody raised against this peptide and the immunohistochemical identification of this peptide and enkephalins in the neuroepithelial cells (NECs) in the gill of several teleost fish species living in different habitats. In spite of the abundant literature on Piscidin1, the biological role of this peptide in fish visceral organs remains poorly explored, as well as the role of the neuropeptides in neuroimmune interaction in fish. The NECs, by their role as sensors of hypoxia changes in the external environments, in combination with their endocrine nature and secretion of immunomodulatory substances would influence various types of immune cells that contain piscidin, such as mast cells and eosinophils, both showing interaction with the nervous system. The discovery of piscidins in the gill and skin, their diversity and their role in the regulation of immune response will lead to better selection of these immunomodulatory molecules as drug targets to retain antimicrobial barrier function and for aquaculture therapy in the future.

## 1. Introduction

### 1.1. Neuro-Immune Interactions in Host Defense

The nervous system, including the brain and peripheral divisions, can either stimulate or inhibit various activities of both innate and adaptative immune systems. Antimicrobial peptides (AMPs) are ancient defense molecules of the innate immune system. Similarly, neuropeptides are ancient signaling molecules. Similarities in size, cationic charge or amphipathic design between some neuropeptides and AMPs suggest that they might serve an additional function in antimicrobial immunity [1]. The interactions of immunomodulatory peptides with human nervous, digestive, lung, cardiovascular and immune system are emerging. For this reason, it is necessary to understand the interaction between peptides and these systems for a better understanding of their function to enhance immune responses, such as lymphocyte proliferation and cytokine regulation, although the specific mechanisms of these activities remain unclear [2]. Several peptides with neural or neuroendocrine signaling functions have been shown to have potent antimicrobial activity. Furthermore, they are widely distributed not only in the endocrine, neuroendocrine and nerve cells, but also in immune cells [1]. These neuro-antimicrobial peptides (NAMP) can be bacteriostatic or bactericidal. There is also increasing evidence that gut neuropeptides are one of the axes of communication between the gut microbiota and the host [3] and they might also be playing a role as antimicrobial agents. Gut neuropeptides are structurally similar to regular antimicrobial peptides (AMPs); they are small molecules and also share similarities with other AMPs in their mode of action. Gut neuropeptides, including gut antimicrobial neuropeptides, form a complex network between the nervous and immune systems [4], where they play a key modulatory role. Gut neuropeptides emerge from enteric neurons in response to different stimuli and also from the enteroendocrine cells [5]. Antimicrobial peptides are also produced by the enterocytes and enteroendocrine cells in the digestive system [6]. In this review, we report the presence of the antimicrobial peptide piscidin 1 and enkephalin previously reported in fish gill neuroendocrine epithelial cells [7,8] as potential regulators that may be able to exert an action by signaling to distant organs, such as the brain, as also emphasized for gut neuropeptides by Aresti Sanz and El Aidy [4].

### 1.2. Interactions between the Immune Cells and the Nervous System in Different Tissues

The interaction of piscidin and gill neuropeptides with the nervous system is still unknown in fish. Neuropeptides actively regulate immune functions in the gut in both direct and indirect ways, allowing for communication between the immune and nervous system. Most of the neuropeptides produced by neurons during the immune response have neuroendocrine functions that can influence both the brain and gut [9]. The nerve structures belonging to enteric nervous system regulate the gut functions autonomously, the remaining extrinsic innervation of the gut consisting of either afferent gut-brain or the efferent brain–gut axis [9]. The gill of fish is an immune-competent organ, as with the gut. In fact, teleost gills are continuously challenged with environmental pollutants/toxins and pathogens, both of which trigger an immune response in teleost gill-associated lymphoid tissue (GIALT) [10,11]. However, several studies have demonstrated that numerous innate and adaptative immune molecules or cells involved in immune-related pathways are present in teleost gills, such as immunoglobulins (Igs), antibody-secreting cells and piscidins [8,11,12,13,14]. Very recently, the mast cells, the mucous cells and the neuroepithelial endocrine cells (NECs) of the gills of teleosts are reported to generate immunomodulatory substances such as piscidin 1 [8,15] that are thought to have an immunomodulatory activity in fish [16]. The gill of fish possesses a branchial innervation that is consistent amongst all fish. Cranial nerve branches V, VII, IX and X innervate the gills, and each arch is innervated by sensory pre-trematich branch and motor/sensory post-trematich branch [17,18]. The gill nerves are a heterogeneous mix of axons since there are both afferent and efferent axons in these nerves and within each of these classes of axons there are multiple sensory receptors (O_2_ chemoreceptors or neuroepithelial endocrine cells (NECs) and effectors (filament muscles and vascular shunts) that have the potential to influence the cardiovascular responses [17] and other physiological functions including a possible immune modulation. In fish gill, no evidence has been produced regarding a communication of its immune cell system with the nervous system, except for a possible mediation by the NECs through afferent-like synapses sending information to central nervous system in hypoxia-induced O_2_ chemoreception [17,18]. Fish skin mucus acts as the first barrier to infections. It is produced in the mucous cells that begin differentiating in the stratum germinativum of the epidermis and migrate to the surface of the skin where they release their contents (see, or review, 70). The epidermis is covered by a glycocalyx or cuticle, consisting of a thin mucus layer that is a complete mixture of molecules derived by the contents of sloughed surface epithelial cells and mucus secreted goblet cells (70,71).The mucus contains lectins, lysozymes, complement proteins, AMPS and immunoglobulins which have an important role in inhibiting the entry of pathogens. Fish skin and gill mucus serve as repository of biologically active substances, as well as numerous defense molecules of both innate and acquired immune system (15,75). The integrity of the skin is necessary for the homeostasis and protecting barrier function. Resident immune cells ensure protection against pathogens and nerve fibers may act on vasculature to induce vasodilation and immune cell recruitment (19).

## 2. The Neuro-Immune Interaction at Barrier Surfaces

### 2.1. Neuro-Immune Circuits in Peripheral Tissues

Barrier tissues, such as the skin and the respiratory tract, are heavily innervated by sensory and autonomic nerves and densely populated by resident immune cells including mast cells, dendritic cells and innate lymphoid cells, allowing for a coordinated response to noxious stimuli. The neuronal release of neurotransmitters, neuropeptides and antimicrobial peptides allows for quick communication with immune cells and their recruitment. The resident immune population ensures both protection against pathogens and maintenance of tolerance against innocuous antigens [19]. Neuroimmune interactions at the lung air barrier surface also occur during lung infections. The neuropeptide calcitonin-gene related peptide (CGRP) mediates the killing of bacterial pathogens. CGRP is also expressed by the pulmonary neuroepithelial endocrine cells (PNECs), in addition to sensory nerves [19]. PNECs were found to play a role in regulating immune cell recruitment in the lungs [20,21]. Recent studies have demonstrated that environmental signals can be sensed at mucosal barriers by neuro-immune sensory units (NICUs): immune and neuronal cells co-localize and functionally interact to steer immune responses [22]. For example, PNECs are seen co-localized with innate lymphoid cells (ILC2). PNECs express gamma-aminobutyric acid (GABA) and also mediate immune functions during allergic airway diseases. PNECs-ILC2 interaction is of remarkable importance in the mucosal neuro-immune interactions in the lung. It is elicited by the secretion of the neuropeptides by the PNECs, which are thought as important regulators of lung immune response [21]. Neuroimmune communication can be essentially regarded as a bi-directional exchange of information that is carried out by classes of molecules, which were originally thought to be restricted to either neural, endocrine or immune systems [23]. These include neuropeptides, monoamines, free radicals and cytokines that were originally described in the central nervous system (CNS) and exhibit a number of immunomodulatory properties. Pro-enkephalin and chromogranin-derived peptides are found in the bovine adrenal medulla. Met-enkephalin represents a unified neuroimmune protective response to an immediate threat to the organism and is envisioned to activate immune cells and to provide a chemotactic signal for further immune cell recruitment [24].

### 2.2. Fish Mucosal Surfaces

The consequence of the above reported observations reported in the mammalian literature and the localization of the enkephalins (met and leu enkephalins) and the antimicrobial peptide piscidin 1 in fish gill neuroepithelial endocrine cells [7,8,25] is the overlap between these endocrines and the immune cells and their interaction with the filament nervous system [15,25]. We argue that the neurotransmitters and antimicrobial peptides released by parasympathetic gill nerves bind to their respective receptors, located on the surface of immune cells and chemoreceptor cells and initiate immune-modulatory responses. Then, a level of cross talk could be mediated by a common currency of signaling molecules. Although data also explaining how the peripheral nerves recognized and responded to bacterial infection—including a possible relation of neural immunity with immune response—are not shown, the presence of the antimicrobial peptide piscidin 1 is reported for the first time in the gill filament peripheral nerves [26,27,28]. This is in agreement with the participation of the mammalian nervous system to a characteristic immune response by producing and releasing AMPs during the repair, allowing the recruitment of peripheral leucocytes and activation of their effector functions [29,30]. This review focuses on the neuro-immune interaction at the gills, which, as with the gut, skin and lung, are densely populated by autonomic vagal nerves, O_2_ chemo sensing cells and immune cells that constantly sense and adapt to tissue-specific environmental challenges [31]. The respiratory and gastrointestinal mucosa maintain an epithelial barrier for commensals, pathogens and foreign antigens and harbor lymphoid tissues and immune cells interacting with neural elements [32]. Our aim is to describe the localization of the piscidin 1 and neuropeptides in fish gill, the air-breathing organs (ABOs) and the skin and discuss the putative function of these signaling molecules in the crosstalk with immune cells, endocrine cells (NECs) and sensory afferents/autonomic efferents [18,32]. Unlike neuroimmune interactions, which are well established and maintained in the peripheral mammalian tissues, this area of investigation is little studied in fish.

## 3. Piscidins and Neuropeptides as a Source of AMPs: Phylogeny, Cell Distribution, Molecular Structure and Immunomodulatory Function

There is an increasing indication that the homeostasis of any organism depends on a constant dialogue with the microorganisms that cover its surfaces, and this also applies to aquatic animals. However, how exactly the formation of a species microbiome is regulated and the mechanisms by which the hosts interact with their microbiome remain largely unknown. All animals, including fish, are constantly exposed to microbes; this applies to their external skin, as well as internal respiratory and gastrointestinal surfaces. Biologically active peptides provide bidirectional interactions between the host tissue and microbiome [33,34,35]. The body compartments of animals are innervated by a diverse network of nerve cells that produce neuropeptides, which serve as messengers in the complex interactions within and between the nerve cells and connected body parts [34,35,36]. Host defense peptides, also known as antimicrobial peptides, belong to a class of molecules expressed predominantly at host–environmental surfaces such as the oral cavity, respiratory tract and gastrointestinal tract. The distribution of these evolutionary ancient molecules supports their antimicrobial function and a key role in signal transmission and modulation in the central and peripheral neural systems [37,38]. In the previous decade, neuropeptides were associated with antimicrobial peptides in that they contribute to the formation of local barriers of defense against pathogens. Notably, inflammatory reactions are regulated by neuronal activity through the production of neuropeptides that cause vasodilation, plasma extravasation and inflammatory cell recruitment [26]. The neuropeptide substance P, calcitonin gene-related peptide, neuropeptide Y, vasoactive intestinal peptide and the antimicrobial neuropeptide PACAP (adenylate cyclase-activating polypeptide) have been demonstrated to have antimicrobial activity in various tissues, including the central nervous system [27,28]. Neuropeptides are now in the spotlight as one of the potential mediators of the exchange of information between the gut bacteria and other tissues and organs [4]. Antimicrobial peptides (AMPS), a family of peptides and proteins with low molecular weight, are present in virtually all life forms, from prokaryotes to eukaryotic plants and animals [39]. These peptides are critical components of the innate immune system in low vertebrate hosts [40]. Piscidins are a subgroup of amphipathic polypeptides [41], which range from 18 to 46 amino acid residues in length. Originally isolated from the striped bass (*Morone saxatilis*), white bass (*M. chrysops*) and their hybrid, there is evidence that piscidins are present in a wide range of teleost fish taxa, including the families Moronidae, Sciaenidae, Siganidae, Belontidae, Cichlidae, Percichthyidae, Latidae, Sparidae, Sygnathidae and Latridae (see, for review, [42]). Piscidin gene transcripts have been cloned as cDNAs and characterized in striped bass and white bass [43], European sea bass (*Dicentrarchus labrax*) [44], Nile tilapia (*Oreochromis niloticus*) [45] and Atlantic cod (*Gadus morhua)* [46]. Piscidins have strong activity against both fish and human Gram-positive and Gram-negative bacterial pathogens [47] and have been localized to mast cells [15,26,48]. As a major class of AMPs, piscidin displays potent broad-spectrum activity against bacteria [48] and parasites [49].

Phylogenetic Inference of Piscidin Genes

**Figure 1:**
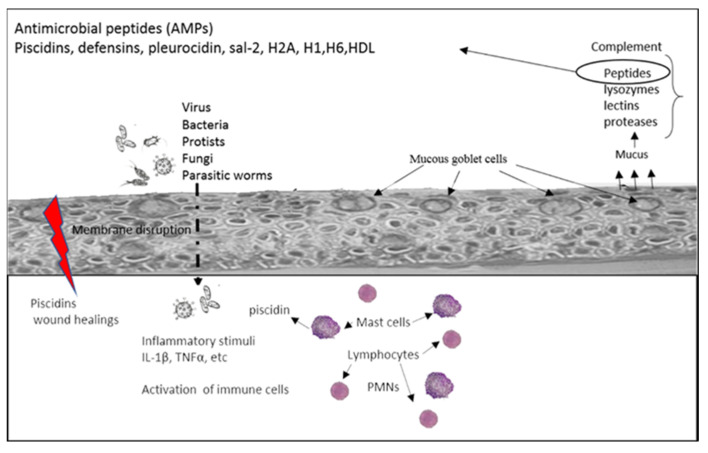
Diagram showing antimicrobial defense mechanism in fish skin. Bacteria, viruses and other pathogens and epithelial cells after skin injuries release cytokines as IL-1β, which attracts neutrophils and T cells to the surface epidermis. Mucus released by the mucous goblet cells is a rich storehouse of AMPS. Some of these (pleurocidin) are involved in immune response to bacteria or opsonize bacteria (cathelicidins). Piscidins are contained in mast cells and mucus and exhibit immunomodulatory and wound healing properties. PMNs, Polymorphonuclear neutrophils. Figure adapted from Rakers et al. *J. Invest. Dermatol.* **2013**, *133*, 1140–1149, https://doi.org/10.1038/jid.2012.503.

Piscidin peptides from basal and evolutionary modern teleost fish are currently characterized in several fish species and have been the focus of a growing body of interest [16,50,51,52]. Unlike other marine vertebrates, which have an external layer of keratinized skin that constitutes an efficient natural barrier, fish are less protected against invading pathogens, since the epidermal surface of the skin is composed mostly of living cells (Figure 1).

**Figure 2:**
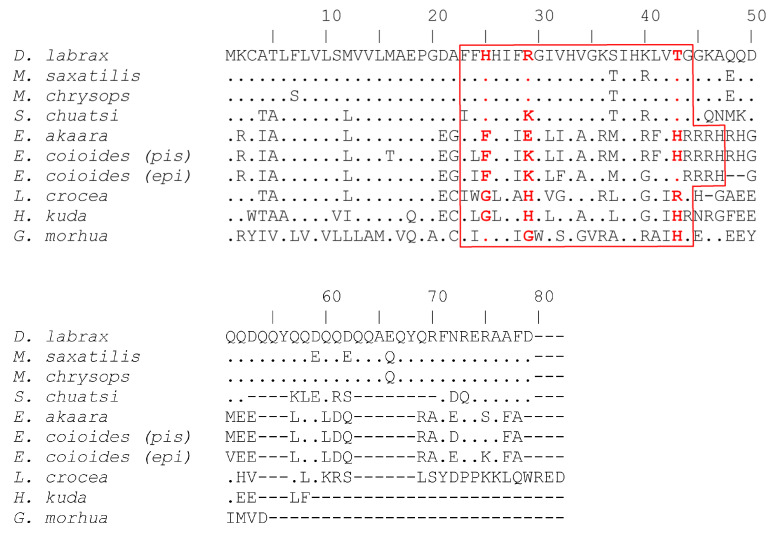
ClustalW multiple sequence alignment of putative piscidin peptides from teleosts. Amino acid residues identical to *D. labrax* piscidin are represented by a dot and alignment gaps are indicated by a dash. The mature peptide is boxed and positively selected residues are highlighted in bold red. Figure from Fernandes et al. 2010 https://doi.org/10.1371/journal.pone.0009501.

**Figure 3:**
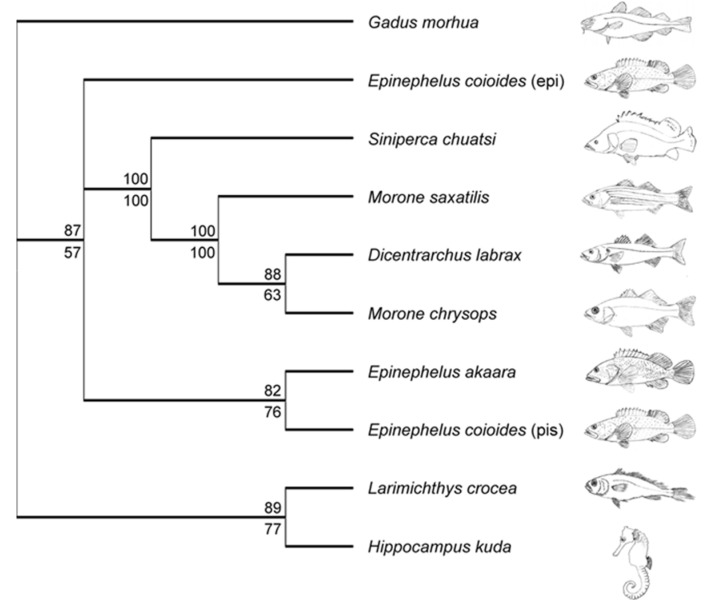
Unrooted rectangular cladogram illustrating the phylogenetic relationship between piscidins. The SYM+G model was selected for the Bayesian analysis and the consensus tree was built after burning 1250 trees from the 5^−10^ generations. The likelihood phylogeny was obtained with a HKY nucleotide substitution model with a discrete gamma distribution (4 categories, gamma shape parameter 2.0) and 100 bootstrap data sets: Bayesian posterior probabilities and maximum likelihood bootstrap values are indicated as percentages above and below the tree nodes, respectively. Figure from Fernandes et al. 2010 https://doi.org/10.1371/journal.pone.0009501.

The piscidin genes of teleosts fish have to cope with the diversity of the capacity of evolving pathogens, and it is therefore likely that they are shaped by positive selection [51]. The nucleotide divergence of piscidin genes was reported recently in the Atlantic cod [51] and likelihood methods with various models of evolution identified patterns of positive selection. Using specific primers, we isolated a 165 bp cDNA, the full length CDS of a piscidin orthologue (Genbank FJ917596). The putative mature cod piscidin is rich in isoleucine (27.3%) and histidine (22.7%) and it contains the two conserved histidines that define the piscidin group of antimicrobial peptides (Figure 2). This result shows that piscidins are an ancient family of host defense peptides not restricted to higher teleosts, since the cod, *G. morhua*, is a basal teleost of the *Paracanthopterygii superorder* [51,52,53]. Most piscidin genes were identified to have a date code for a precursor comprising a 22-residue signal peptide, a mature (active) peptide of 22–25 residues and a variable C-terminal region. The *G. morhua* sequence is very different from any other fish species and amino acid identities with its orthologues ranging from 27.2% to 36.3%. This marked divergence may be explained by the longer evolutionary distance between *G. morhua* and other fish. The mature piscidin is generally less conserved across the different taxa, sharing only 31.8% to 95.4% of its identity between species and amino acid level (Figure 2). A difference in the conservation levels amongst various domains is common amongst AMPs. The cathelicidins are only recognized by their conserved signal sequence, since the mature peptides are diverse in length, amino acid sequence and even secondary structure. Both maximum likelihood (ML) and Bayesian inference produced a phylogenetic tree with high credibility support (Figure 3).

**Figure 4:**
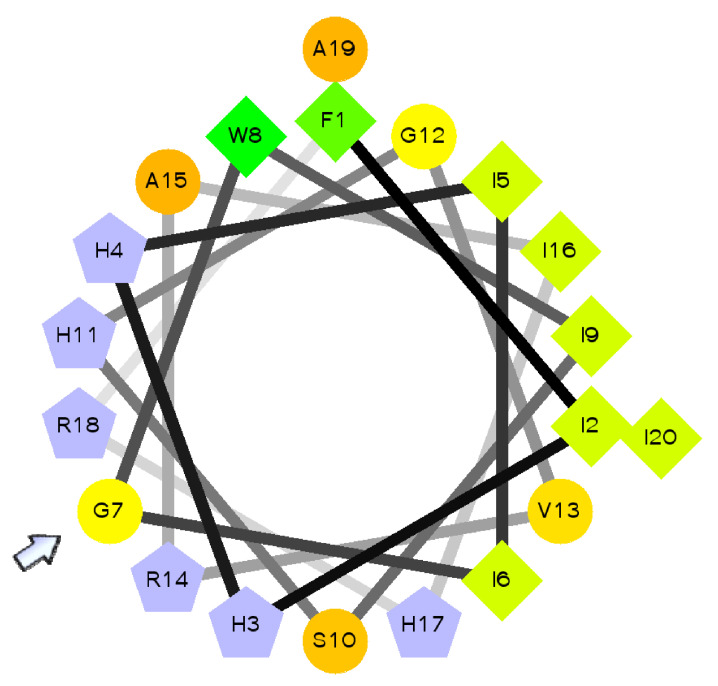
Amphipathic alpha-helical structure of cod piscidin. Schiffer–Edmundson helical wheel projection of piscidin from *Gadus morhua*, showing that it is predicted to adopt an almost perfect amphipathic structure. The amphipathicity of the helix is disrupted by glycine at position 7 (arrow), which corresponds to a positively selected site. Hydrophilic charged and very hydrophobic residues are represented by grey pentagons and green diamonds, respectively. The circles denote other neutral and polar amino acids. Residues are numbered starting from the amino terminus of the mature peptides. Figure from Fernandes et al. 2010 https://doi.org/10.1371/journal.pone.0009501.

In general, the topology of this tree is in accordance with various fish. The exceptions are *L. crocea* and *H. kuda* piscidins, which cluster together and separately from Acanthopterygii. All the positive selected sites in the piscidin gene are located within the region corresponding to mature peptide. The higher positive selection sites net charge at physiological pH and the amphipathic nature of piscidin 1 are thought to be crucial for their ability to permeabilise bacterial membranes [54,55]. Several amino acid substitutions observed in positively selected sites within the mature peptide change its charge or amphipathicity. In particular, site 29 in *G. morhua* piscidin corresponds to glycine that disrupts the amphipathic nature of the peptide (Figure 4), which in turn is associated with adaptation of piscidin to pathogens in new ecological niches [51]. In this scenario, it may be taken into account that many duplicated piscidin genes retain their original antibacterial functions, other genes are retained owing to different processes, particularly subfunctionalization, or neofunctionalization, by gaining a novel function. Piscidins that are subjected to these evolutionary processes result in a diversified family with different anti-bacterial and anti-protozoan properties [43,54,56]. As reported above by Salger et al. [42], it appears that the different groups of piscidins may differ based on their peptide structure and size, phylogenetic analysis, gene expression and antimicrobial activity, and have different functions in the fish innate immune system. Knowledge of piscidin function and activity may help for the use of the innate immune system to control disease-related mortality in aquaculture and a novel alternative to conventional drugs [57]. Table 1 and Table 2 show a list of the different isomers of piscidis, their sequence, molecular weight and their properties, as well as a list of pathogens, their expressions ites, organisms and up- and down-regulation of piscidin genes.

## 4. Neuropeptides and AMPs and Immune-Associated Function

Neuropeptides, cytokines and hormones have been identified as signaling molecules mediating the communication between three systems: the nervous, endocrine and immune system [23] (Figure 4).

Some AMPs have been shown to be derived from the enzymatic processing of neuropeptide precursors, such as the proenkephalin A (PEA) and chromogranin B, a key mechanism to generate an antimicrobial defense in tissues [75,76]. PEA is a precursor of the enkephalin opioid peptides, proteolytically processed to yield several biologically active peptides, including met-enkephalin and leu-enkephalin. These processing enzymes are expressed in leucocytes, as in the neuroendocrine system [76]. Both the AMPs and neuropeptides highlight the known mechanisms by which the nervous system can influence immune function. Due to the similarities in amino acid composition, amphipathic design, cationic charge and size, it has been suggested that antimicrobial peptides, many neuropeptides and peptide hormones may be directly involved in innate immune reactions against pathogen intruders [76]. In mammals, neuropeptides, such as substance P, calcitonin-gene related peptide (CGRP), Neuropeptide Y, vasoactive intestinal polypeptide (VIP), somatostatin and enkephalins, have all been suggested as participating in the bidirectional organ–brain communication [36,76]. However, the role in the interaction with the nervous system is still yet to assessed. Our study revealed that enkephalins and piscidin colocalize in the gill NECs and the nervous system of the gill filaments of fish, playing a possible role in bidirectional relationship between the host and its associated microbiota [77].

## 5. Neuroimmune Communication in the Gill-CNS Axis

The field of neuroimmunology both within the CNS and the periphery is currently one of the most exciting fields in immunology and remains poorly investigated in fish. Current mammalian studies mainly focus on multiple direct and indirect pathways that maintain intensive and extensive bidirectional interactions between the gut microbiota and the CNS, involving endocrine, immune and neural pathways [78], forming a basis of the so-called MGB axis (microbiota–gut–brain axis) [77]. Over the course of evolution, the microbiota established important feedback channels with CNS, some of which are important for maintaining homeostasis [79]. In the MGB axis, lymphocytes may sense the gut lumen and internally release cytokines, which may have endocrine or paracrine actions; sensory nerve terminals, such those of vagus nerve, may be activated by gut peptides released by enteroendocrine cells. The communication processes are based on neurotransmitters, neuropeptides, cytokines, hormones and growth factors that establish the relationship between the immune system and the CNS, leading to tissue homeostasis [80]. In agreement with what was highlighted by Das and Salinas [81] regarding the neuroimmune interactions in the fish olfactory organ and those governing the immune responses in the rest of all mucosal tissues of teleosts, we found that the gill, as with the olfactory organ, is also an important route for pathogen entry into fish, based on the immunohistochemical evidence of immunoregulatory substances in the immune cells, sensory nerves and neuroepithelial endocrine cells [8,15,26]. For example, the intraepithelial localization of eosinophils, their interaction with mast cells and the interaction of mast cells with sensory nerves and the occurrence of mast cells in the gill surface layers and their opening in the exterior environment [15,25]. Some findings show the further presence of a lymphoid tissue [10] protecting the gill from water borne pathogens. The association of mast cells with gill sensory nerves is a microenvironment different from that of the neuroepithelial endocrine cells (NECs) expressing piscidin [8]. These cells were seen adjacent to both Pis-positive mast cells and Pis-positive nerve terminals, thus constituting a different microenvironment which promotes neuroimmune communication. In fish, the nerve trunks connecting the gills to the brain are branches of cranial nerves, especially the glossopharyngeal and vagal nerves [82]. A substantial fraction of the nerves running the branchial trunks carry afferent sensory information from the NECs within the gills. A striking similarity between the nervous system and the immune system lies in their ability to sense environmental cues [81]. Although these systems were believed to work independently, many studies [83,84] show the crosstalk between neurons and immune cells, both in the CNS and periphery. Some examples include the release of neuropeptides by danger-activated nociceptor neurons that regulate innate and adaptative immune responses [85]. We have no evidence for a neuroimmune interaction at fish mucosal surfaces, except for that in the olfactory organ [81].

## 6. Neuroimmune Cell Crosstalk in the Gill

**Figure 5:**
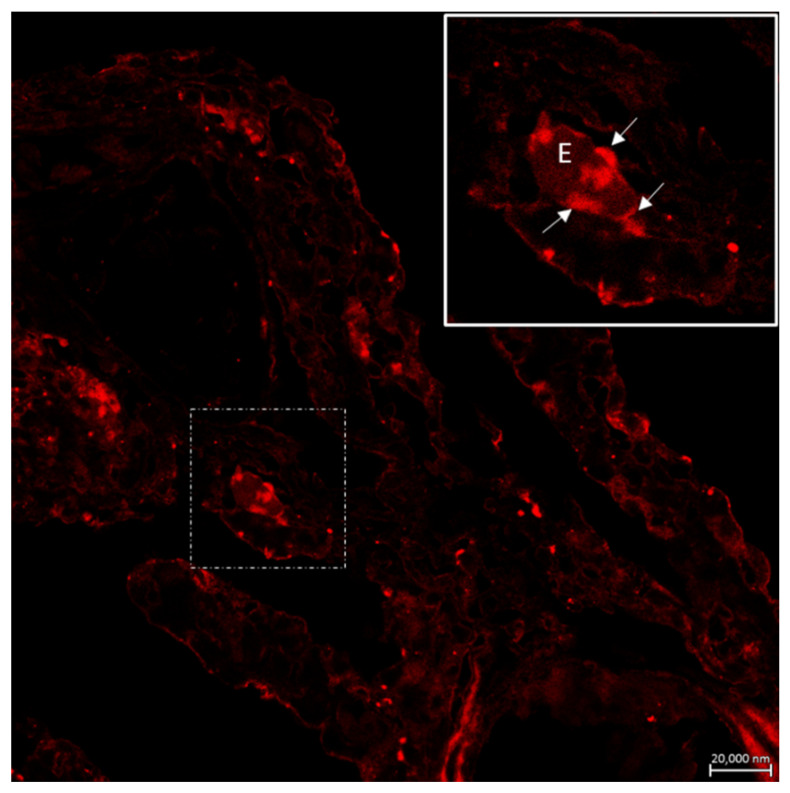
Confocal image of the gill filament of *Pantodon bucholzi*. Immunostaining with antibody against Piscidin 1 (Pis 1). Co-localization of one eosinophil (E) with mast cells (arrows and inset), the latter showing highest immunoreactivity for Pis 1. Scale bar = 20 µm. Figure from Capillo et al. 2021 https://doi.org/10.1016/j.fsi.2021.02.006.

**Figure 6:**
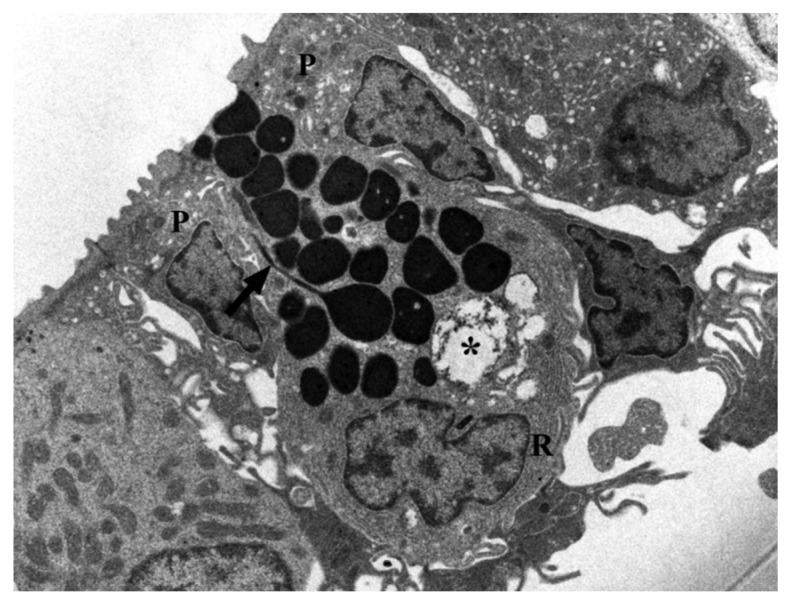
A polarized mast cell of the gill of *Clarias gariepinus* reaching the external environment between pavement cells (P). Endoplasmatic reticulum (R). Several cytoplasmic granules appear empty (*asterisk). Another granule shows a tubular elongation (arrow) that may reach the surface for secretion. Scale bar = 2 µm.

**Figure 7:**
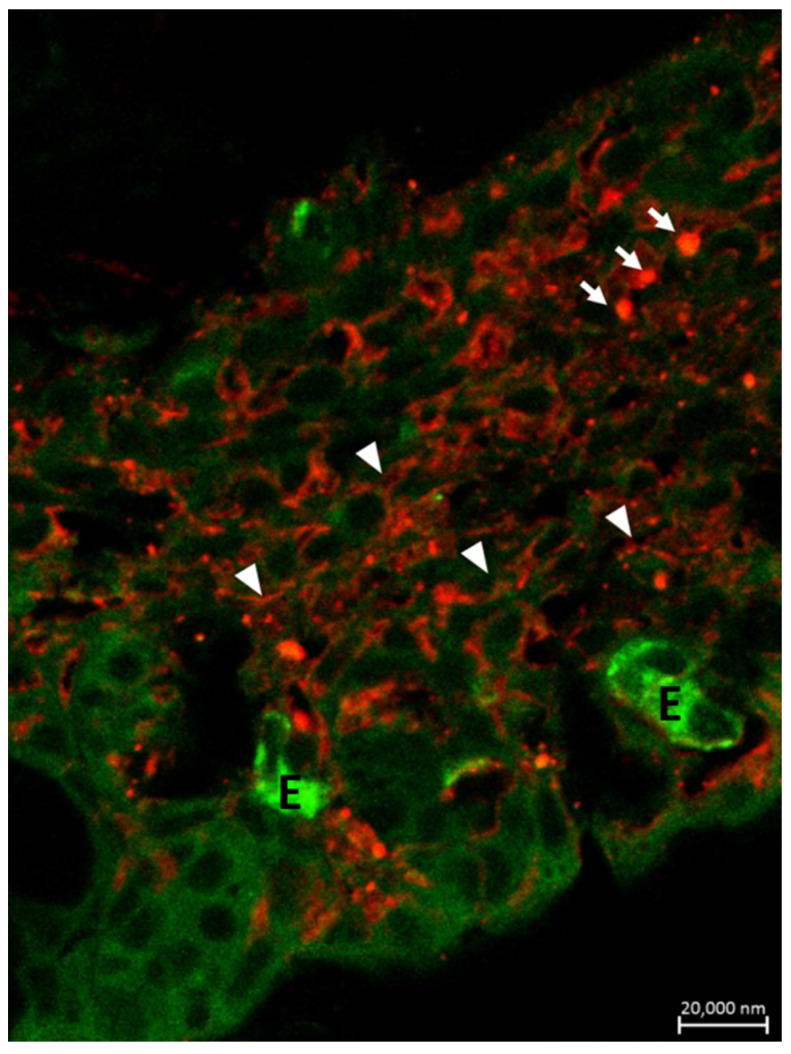
Immunohistochemical demonstration of mast cell nerve interaction in the epithelium of the suprabranchial chamber of *Clarias gariepinus*. Section stained for Pis 1 (in red) and 5HT (in green) showing the close anatomical association of Pis-positive nerves (arrowheads) with Pis-positive mast cells (arrows). Eosinophils (E) showing 5HT immunoreactivity. Scale bar = 20 µm.

Mast cells have a widespread distribution and are found predominantly at the interface between the host and the external environment. Mast cell maturation, phenotype and function are a direct consequence of the local environment and have a marked influence on their ability to specifically recognize and respond to various stimuli through a release of an array of biologically active mediators [86]. The versatility of mast cells provides them the potential to not only act as first responders in harmful situations, but also to react to environmental changes by communicating with a variety of other cells implicated in physiological and immunological responses. Mast cell phenotype is dictated by the microenvironment and animal species. Mast cells are found near externally exposed surfaces such as the epithelium of the skin, the gastrointestinal tract and airways [87]. We have found that mast cells in the gills of our fish species co-localize with eosinophils in *Pantodon bucholzi* [15] (Figure 5). In the gills and suprabranchial chamber of the African catfish *Clarias gariepinus*, Pis-immunopositive mast cells are seen in close anatomical association with Pis-positive sensory nerves [25]. Some mast cells are also seen in the outermost cell layer of both gill (Figure 6 and Figure 7) and air-breathing organs (ABOs) epithelia. This proximity to the external environment makes mast cells prime candidates to mount rapid responses to external stimuli and the internal microenvironment.

**Figure 8:**
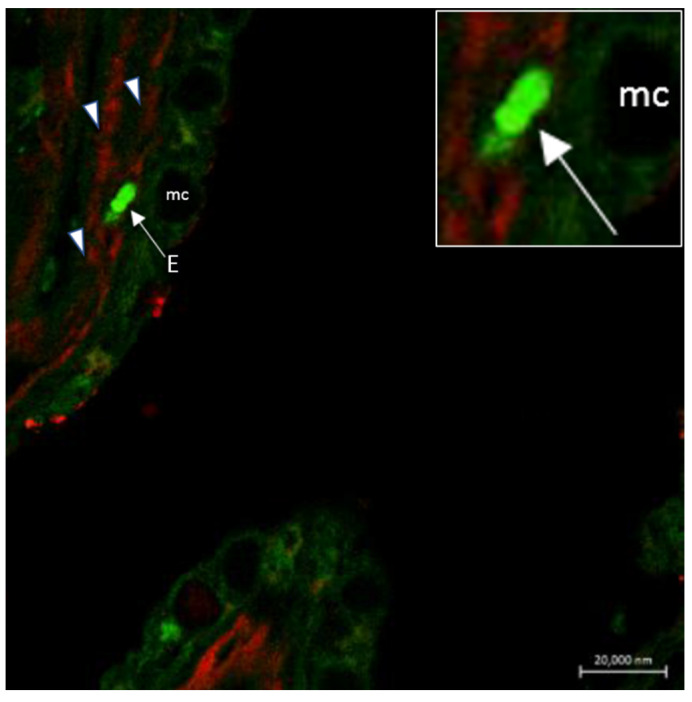
Immunohistochemical demonstration of eosinophil nerve interaction in the gill of *Heteropneustes fossilis*. Section stained with antibodies against 5HT (in green) and Pis 1 (in red) showing the presence of a subepithelial eosinophil (E) stained with 5HT antibody in close proximity to Pis-positive nerve terminals (arrowheads). MC = Mucous cells. Scale bar = 20 µm.

**Figure 9:**
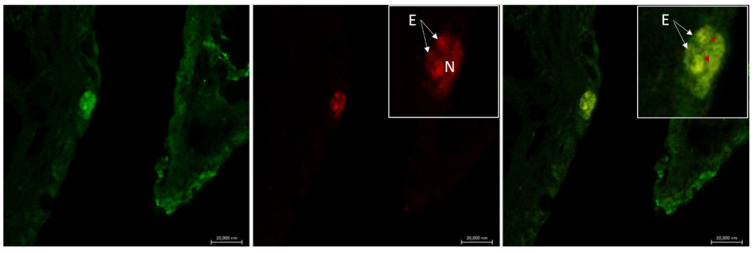
Immunohistochemical demonstration of eosinophil nerve interaction in the gill epithelium of *Heteropneustes fossilis*. Section stained with antibodies against 5HT (in green) and Pis 1 (in red). Endings of extrinsic nerves (N) are in close proximity to eosinophils (E) as shown in red channel and merged image (red arrows). Scale bar = 20 µm.

**Figure 10:**
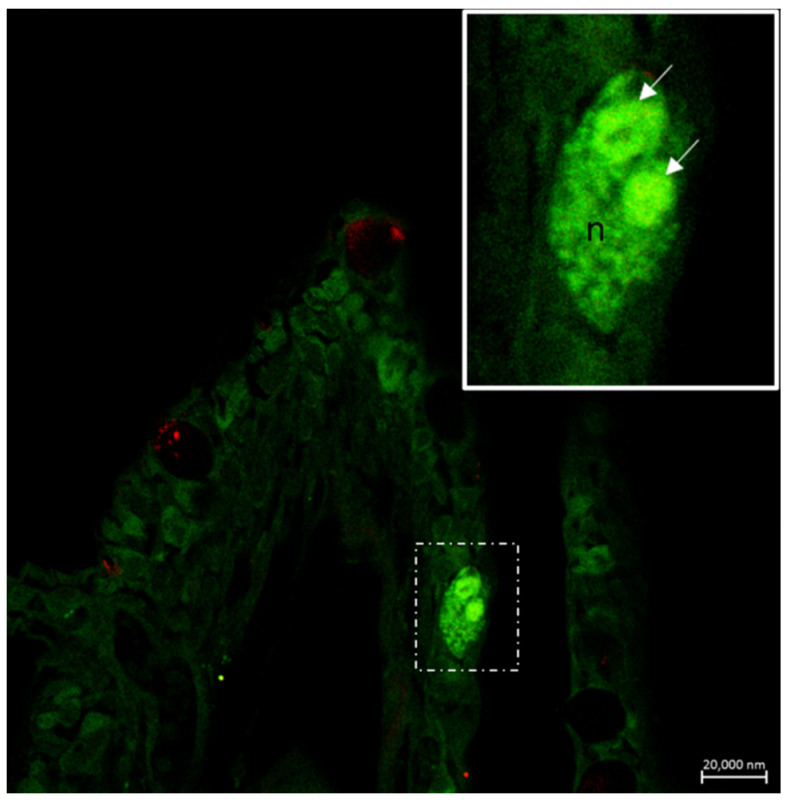
Neuroeosinophilic co-localization in the gill epithelium of *Heteropneustes fossilis*. Section immunostained with antibodies against GABA B R1 and 5HT. The merged image shows in the inset two eosinophils localized to nerves (n). Scale bar = 20 µm.

Eosinophils are seen in both epithelial and subepithelial localization and the blood vessels in the gills of *Heteropneustes fossilis* and *Clarias gariepinus* and in close contact with Pis and 5HT immunopositive nerve fibers (Figure 8 and Figure 9). They also are immunolabeled with antibodies to GABA B R1 receptor (Figure 10).

**Figure 11:**
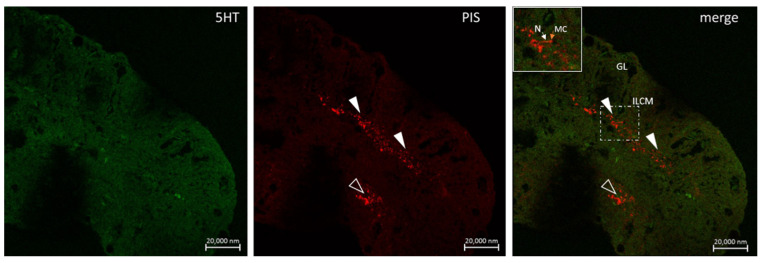
Neuron-mast cell co-localization (arrowheads) in the gill of *Heterotis niloticus*. Section immunostained with antibodies against 5HT and Pis 1 showing the covering of the gill lamellae (G, merged) by a mass of cells termed, the interlamellar cell mass (ILCM). Note the close association of a mast cell (MC) with nerves (N) in the inset of a region of the merged image. Scale bar = 20 µm.

**Figure 12:**
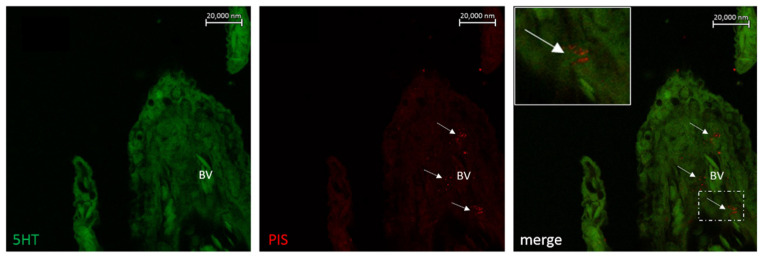
Section of the gill of *Boleophthalmus pectinirostris* immunostained with antibodies against 5HT and Pis 1 showing the presence of Pis-immunopositive mast cells in subepithelial localization and blood vessels (BV, arrows). Scale bar = 20 µm.

**Figure 13:**
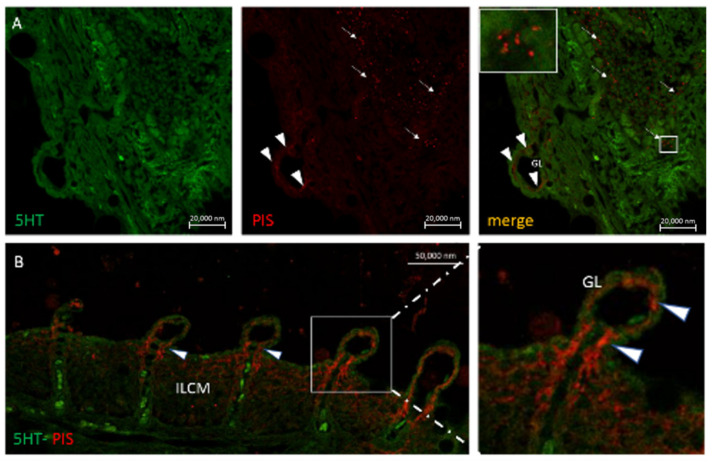
(**A**,**B**). Section of the gill of *Heterotis niloticus* immunostained with antibodies against 5HT and Pis 1. Arrowheads and inset in (**B**) mark the presence of Pis-immunopositive nerve fibers around the gill lamellae and long arrows in (**A**) pick the localization of mast cells in subepithelial smooth muscle (inset and merge). GL, Gill lamellae; ILCM, Interlamellar cell mass. Scale bars = 20 µm (**A**). Scale bars = 50 µm (**B**).

In the gill lamellae of *Boleophthalmus*, the eosinophils are immunostained with the antibodies against Pis 1 and 5HT and make contact with nerves labelled with antibodies against acetylated tubulin. In the subepithelium of the gill filament of this fish, some Pis-positive mast cells are seen in close vicinity with sensory axons located by tubulin antibodies, as well as within the efferent filament artery. In *H. niloticus* gills, we demonstrate the presence of Pis immunopositive mast cells receiving neuronal Pis-positive innervation at the base of the gill lamellae and in the interlamellar cell mass (ILCM), filling the gill filament interlamellar channels; the leading edge of the lamellar epithelium, where mast cells are also co-localized with 5HT; and subepithelial smooth muscle. Pis-positive perivascular innervation is seen also around the blood pathway of the outer channels of the gill lamellae. Pis-immunopositive mast cells are also seen in the subepithelial smooth muscle cells of the gill filament (Figure 11). In *B. pectinirostris*, Pis-immunopositive mast cells are localized near and within the main vasculatures, the intermediate and basal cell layers and near the mucous cells in the gill filaments (Figure 11, Figure 12 and Figure 13A,B)).

**Figure 14:**
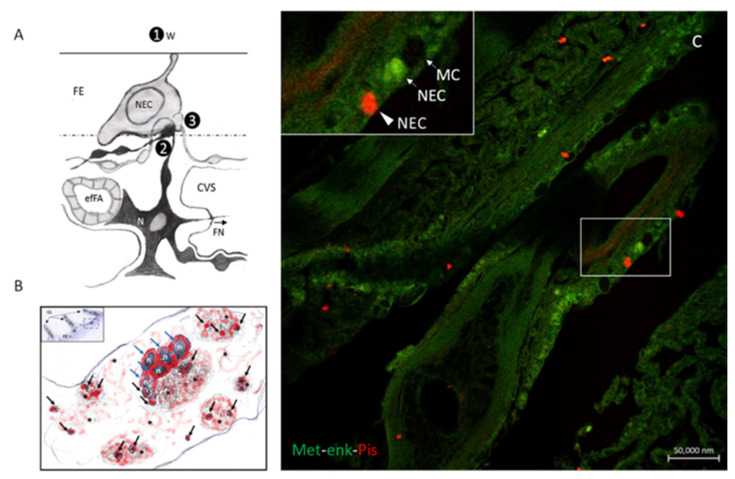
(**A**–**C**). Diagram of the interaction between NECs and mast cells and the innervation of the NECs in the gill filament. (**A**). 1, External environment; 2, afferent synapses and the 5HT neurons (N) connect to central nervous system through the filament nerve (FN); 3, afferent synapses on sympathetic and cranial (parasympathetic) nerves, efFA, efferent filament artery, CVS, central venous sinus. Figure adapted from Bailly (2009). (**B**). Scheme of the interactions among the NECs, mast cells and nerves from a gill section of the catfish *H. fossilis* immunolabeled with antibodies to Pis 1. NECs (blue arrows) are innervated by afferent vagal nerve endings (asterisks) and are in close contact with mast cells (black arrows, Pis 1: red fluorescence). These cells’ release, in addition to Pis 1, 5HT, neuropeptides, GABA and its receptor GABA B R1, also contains acetylcholine and its muscarinic and nicotinic receptors, and regulate both innate and adaptative mucosal immunity. (**C**). Section of the *H.fossilis* gills immunostained with antibodies against Met-enk (green) and Pis 1 (red), showing the presence of Pis-positive NECs (arrows) and NECs immunostained by both the two antibodies (arrowheads). Scale bar = 20 µm.

**Figure 15:**
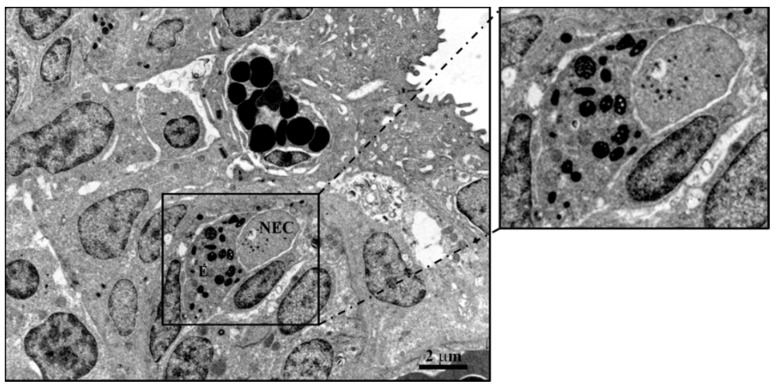
Ultrastructural details of air-sac of *Heteropneustes fossilis*. An eosinophil with granules appears closely associated to a NEC. Scale bar = 2 µm.

The immune cells express neuropeptides, their receptors and components of the GABA-ergic system, including GABA receptors, GABA transporters and GABA metabolic enzymes. The non-proteinogenic amino acid GABA acts as an intercellular signaling molecule in the immune system and as an interspecies signaling molecule in host–microbe interactions [88,89]. In the gill of the Indian catfish *Heteropneustes fossilis*, we found a co-localization of mast cells and NECs, both immunoreacting to the peptide antimicrobial peptide Pis 1 (Figure 14). In this species, we report for the first time the interaction of Pis 1 positive mast cells with Pis 1-positive NECs and the interaction of NECs with Pis 1-positive associated innervation (Figure 14). Eosinophils showing immunoreactivity for both Pis 1 and 5HT are seen in close contact with NECs, as also demonstrated via transmission electron microscope (Figure 15).

Thus, eosinophils expressing GABA B R1 and NECs containing immunoreactivities for both GABA B R1 and GAD67, the latter being the principal GABA synthesizing enzyme [8], may have a high impact in the GABA signaling machinery, although the precise subunits constituents of GABA R subunits remain uncharacterized in fish. In *B. pectinirostris*, close to numerous large 5HT positive NECs in the apical end of the gill filament, and found in the extracellular spaces of these cells, numerous Pis-positive mast cells were observed. Few NECs show Pis immunoreactivity and are seen in the intermediate and apical cell layers. In the ILCM of the gill filament of *H. niloticus*, some 5HT positive NECs are seen in close contact with Pis-positive mast cells. It is important to keep in mind that a given profile of mediators (not determined in our fish species) may vary considerably according to the species studied, subtype and surrounding microenvironment where mast cells are found. Mast cells are localized in association with the peripheral nervous system and the brain, where they are also aligned, anatomically and functionally, with neurons and neuronal processes throughout the body [90]. As a neuroimmune archetype mast cells participate in mammals as an essential intermediary between the immune and nervous systems. Mast cells express receptors for various classical neurotransmitters, including acetylcholine (Ach), the gaseous neurotransmitters (NO), calcitonin-gene related peptide (CGRP), opioid peptides(enkephalins) and other neuromodulators. Mast cells are found in sites where they launch a first line of defense by releasing AMPs [87]. The authors studied the molecular interactions of piscidins that activate chemotaxis of other immune cells (neutrophils) through the presence of formyl peptide receptors. It was recently reported in Nile tilapia that Piscidin 4 can also reprogram M1 macrophages to M2 phenotypes through the MAPK/ERK pathway and IL-10-STAT3 signaling [91]. In our studied fish species, we found a close interaction of mast cells with nerves and NECs (see above), but also a closeness of mast cells with eosinophils showing immunoreactivity for Pis 1 antibody, nitric oxide (NO) and tyrosine hydroxylase (TH) and 5HT [25]. However, the molecular interaction of mast cells with the diverse immune cells, including the NECs and host-defense molecules, is not known and knowledge of this may help to inform the design of the more complex interactome of immune cells.

### 6.1. Neuropeptides

**Figure 16:**
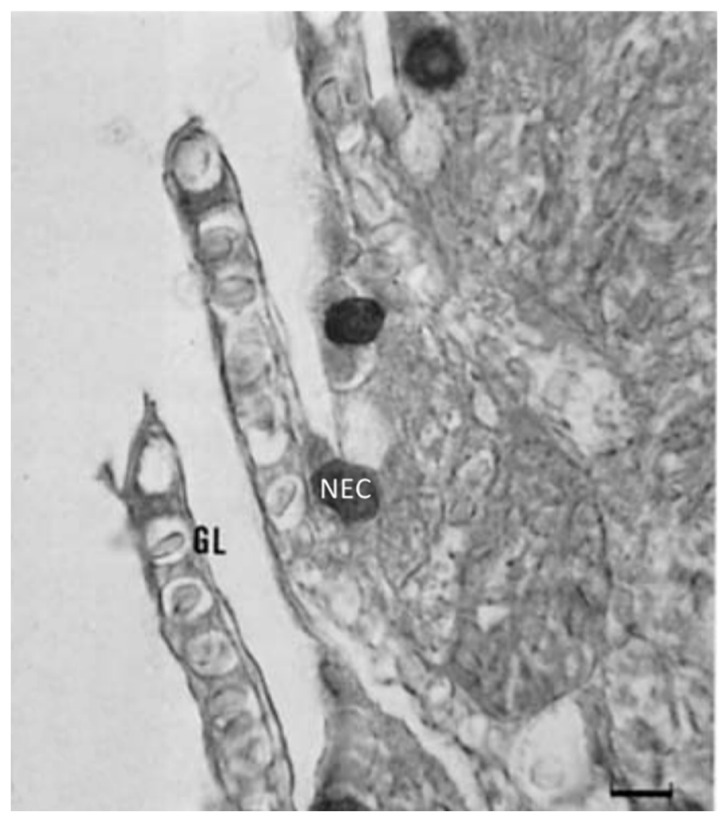
Met-5-enkephalin immunopositive NECs lie in the multi-layered filament epithelium of trout. Scale bar = 20 µm. Figure from Zaccone et al. 1992 https://doi.org/10.1111/j.1463-6395.1992.tb01185.x.

**Figure 17:**
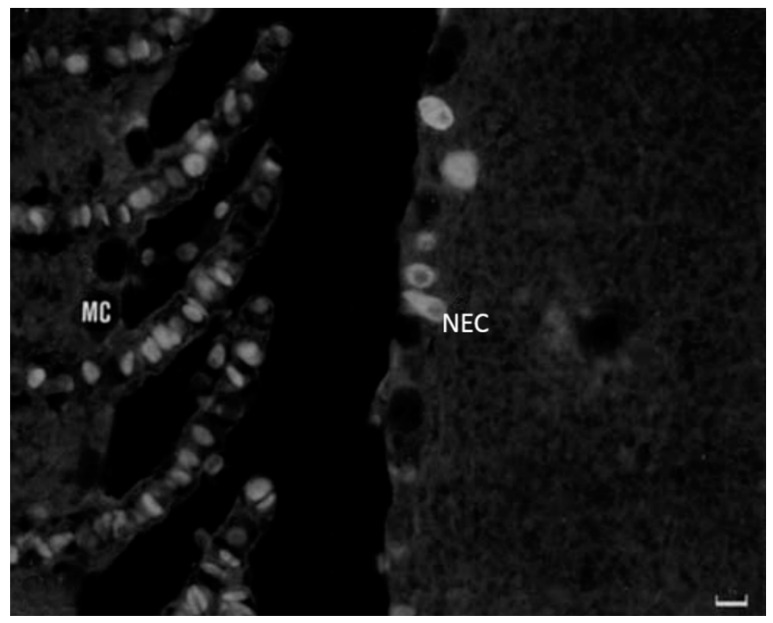
Leu-5-enkephalin immunoreactive NECs are seen in the leading edge of the gill filament of trout. Scale bar = 20 µm. Figure from Zaccone et al. 1992 https://doi.org/10.1111/j.1463-6395.1992.tb01185.x.

**Figure 18:**
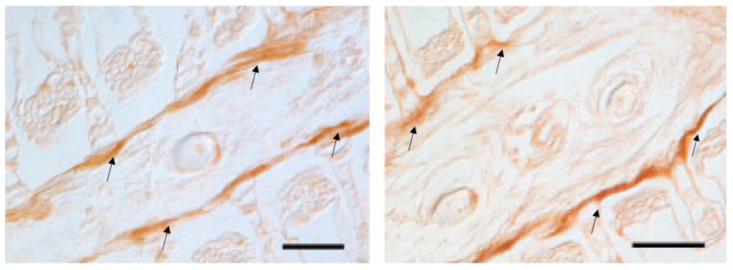
Met-5-enkephalin positive nerve bundles (arrows) are located beneath the basal lamina of the gill filament of *Pangasius sutchi*. Scale bar = 20 µm. Figure from Zaccone et al. 2006 https://doi.org/10.1002/jez.a.267.

Studies on immunoreactivity for regulatory peptides have been conducted on fish gill epithelia by Zaccone et al. [7]. The authors described two distinct populations of NECs that exhibited immunoreactivity either for 5HT or for met-enkephalin and leu-enkephalin. The immunoreactivity for enkephalins does not coexist with the immunoreactivity for 5HT. The second population of cells reacting positively to met-enkephalin and leuenkephalin is reaching the epithelial surface (Figure 16 and Figure 17). Met-enk immunoreactivity is contained in thick nerve bundles located beneath the basal lamina of the gill filaments (Figure 18). We observed the colocalization of these neuropeptides and Pis in numerous NECs and the presence of only Pis-positive NECs, both along the filament epithelium and in the apical end of the gill epithelium of the catfish *H. fossilis* (Figure 14C).

### 6.2. Neuro Mast Cell Crosstalk in the Skin

**Figure 19:**
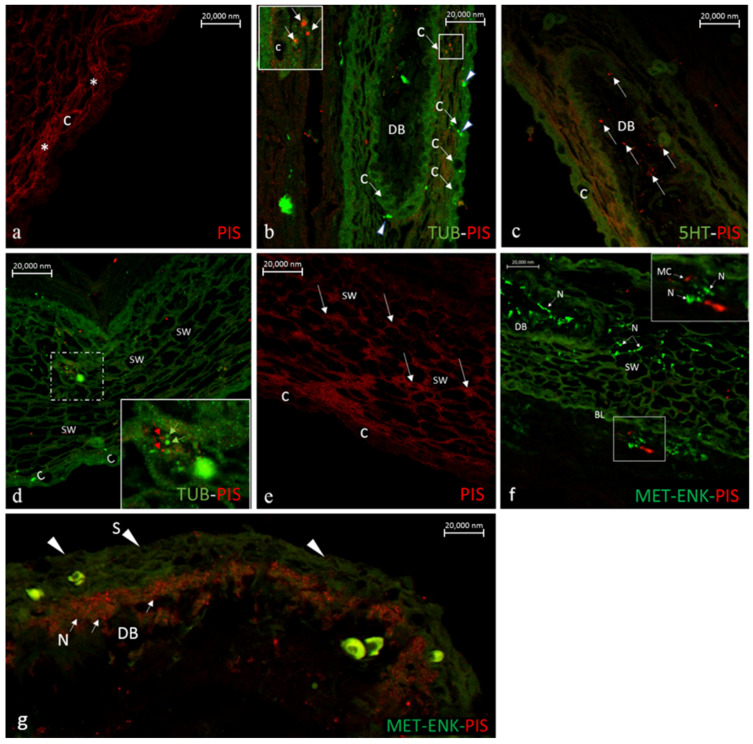
(**a**–**g**). (**a**). A dense network of Pis 1 positive nerve fibers (asterisk) is running in the capillaries (C) located in the head epidermal surface of *Boleophthalmus pectinirostris*. (**b**). Longitudinal section of the head epidermis of *B. pectinirostris* stained with antibodies against tubulin and Pis 1. Numerous sensory axons (arrowheads) immunolabeled with tubulin antibodies are seen in close contact with capillaries (long arrows). Pis-positive mast cells (inset) are seen in close contact with a capillary (C) lying at the apex of a dermal bulge (DB) or respiratory papilla. (**c**). Longitudinal section of a dermal bulge (DB) in the ventral skin of *B.pectinirostris* stained with antibodies against Pis 1 and 5HT showing the presence of mast cells (long arrows) in the center of dermal bulge (DB). (**d**). Longitudinal section of the head skin epidermis of *B.pectinirostris* immunolabeled with antibodies against tubulin and Pis 1 showing mast cell (red arrows) interaction with tubulin positive sensory axons (green arrows). C, Capillaries; SW, Swollen cells. (**e**). Longitudinal section of the head skin epidermis of *B. pectinirostris* immunolabeled with antibodies against Pis 1. The dense network of Pis-positive nerve fibers around the swollen cells (SW) of the intermediate epithelium and the capillaries (C) at the outermost surface of the skin are seen. (**f**). Head skin immunolabelled with antibodies against Pis 1 and met-enk showing the presence of met-enk positive nerve fibers (N) in the center of dermal bulge (DB) and around the swollen cells (SW) in the intermediate epidermis.Note in the subepithelium the site contact of Pis-positive mast cells (in red) with met-enk positive nerve fibers (in green). (**g**). Head skin immunolabelled with antibodies against met-enk and Pis 1 antibodies showing a rich Pis-positive innervation (arrows) of the capillaries at the apex of a dermal bulge (DB). Scale bars A–E = 20 µm.

Mammalian and fish skin share protective activities against environments that are rich in infectious agents. Fish epidermis is endowed with an extrinsic barrier consisting of a mucus layer and antimicrobial peptides [92]. Recent findings point to the mechanisms through which pathogens interact with each other on the skin and the signaling systems that mediate the communication between the immune system and skin-associated pathogens. The skin is exposed to the modification of the external environments and actively regulates the microbial colonization and microbial entry into dermis/subcutis. Microbes interact each other and with host cells, including the keratinocytes and immune cells, in turn influencing the skin homeostasis. Skin microbiota can inhibit the colonization of pathogenic microbes that stimulate keratinocyte antimicrobial peptide production and inhibit inflammatory cytokine release and inflammation during wound healing [93]. Although the specific ecological networks regulating the activity of the fish skin-associated microbes, as well as immune-microbe crosstalk, are not yet known, the presence of mast cells in fish epithelia is well documented and their production of piscidins in a wide variety of teleost species. Mast cells in mammalian skin are located in the upper epidermis and contribute to maintaining microbiome-tissue homeostasis [93]. They produce antimicrobial peptides and have bactericidal activity. Mast cells recognize microbes through different mechanisms, including the direct binding of pathogens or their components to Toll-like receptors and activation of complement receptors [93]. Studies on fish mast cells, their degranulation and contribution to promoting innate and adaptative responses are, however, not available, including their interaction with the peripheral nervous system. In addition, regarding the gill mast cells, our recent observations in the skin of the mudskipper *B. pectinirostris* have shown the immunolocalization of Piscidin 1 in the mast cells and sensory nerves. Combined immunolabeling procedures using an array of antibodies to 5HT, Pis 1 and acetylated tubulin have demonstrated a rich innervation of the capillary networks that are present near the surface of the epidermis and the dermal capillaries in the apex of the so-called dermal bulges or respiratory papillae (Figure 19a,b,g). Pis 1 immunoreactivity was noticed in the mast cells, which was also found in the center of the respiratory papillae (Figure 19c) and the outermost cell layers of the skins. Axon bundles positive to tubulin antibodies are seen opposed to the capillaries in the skin surface (Figure 19b). In mid-epidermis are seen Pis 1 immunopositive mast cells in closest vicinity to sensory axons immunolabelled by tubulin antibodies (Figure 19d) and in the subepithelium, Pis-positive mast cells are approaching met-enk positive nerve fibers (Figure 19f), thus indicating the cross talk between the mast cells and sensory nerves. The physiological relevance of these signals, however, remains to be established. The swollen cells in the intermediate cell layers of the epidermis are also associated with sensory innervation, as demonstrated by d immunolabeling methods with Pis 1 antibodies (Figure 19e).

## 7. Conclusions

The increasing knowledge about mucosal barriers (gut, lung, skin)–brain bidirectional communication has provided scientific evidence that this system in mammals involves multiple pathways with the participation of neural, endocrine and immune cells. The existence of a close communication between afferent nerve terminals and immune cells (e.g., eosinophils and mast cells) within the gill and ABO epithelia in fish has been established in this review and previous reports [8,15,26]. For example, the terminals of afferent nerve terminals contain a variety of neuroactive compounds and AMPs that are secreted by mast cells and eosinophils, such as 5-HT, piscidin 1, nitric oxide (NO), tyrosine hydroxylase (TH) and the nicotinic acetylcholine receptor. Furthermore, neuropeptide receptors, such as the GABA B R1, have been identified in eosinophils and NECs, thus suggesting a close communication between the nervous and immune system [94]. Neuroepithelial endocrine cells (NECs) constitute a large population of the gill epithelia [95]. Different NEC populations were identified according to the type of bioactive molecule and immunomodulatory substances. These cells are regarded as hypoxia-sensitive chemoreceptors in fish, initiating the cardiorespiratory reflexes, but also acting as immunomodulators in the lung [96] and communicating with the CNS via paracrine signals to vagal afferents [18]. NECs have been long thought to act as both pre-synaptic and post-synaptic elements in the fish gill [18]. Thus, we also propose that an immune and endocrine pathway may also be a route of the gill–CNS axis, although no studies are currently available regarding the involvement of the NECs in the inflammation. However, reports on the NECs in mammalian lung have highlighted their participation in the recruitment of these cells in immunomodulation [21]. AMPs can be derived from the enzymatic processing of neuropeptides and recent molecular investigations [51] demonstrated that they are an ancient family of host defense peptides not restricted to higher teleost fish. It is likely that fish AMPs are not only powerful endogenous antibiotics—as with the piscidins—with a broad spectrum activity, but they also display roles such as the regulation of inflammatory and immune responses [97]. We have also yet to realize the potential benefits that the discovery of piscidins in fish and their diversity and characterization might offer to biotechnology for their development as drugs in biomedicine and to combat fish bacterial infections in aquaculture [68,98].

### Future Directions

As with terrestrial vertebrates, fish have a local immune system that protects the host against invading pathogens. We highlight, in this review, the interaction between the peripheral immune and nerve systems in the skin and gill, and predict that these neuro–immune interactions govern immune responses at all mucosal tissue in fish. Aspects of fish mucosal immunity are now focused to mucosal vaccines and neuroimmunology, and much progress has been made towards understanding how fish respond locally and systemically to infections and vaccination [81].

## Figures and Tables

**Table 1:** List of different isomers of piscidins, their sequence and their anti-viral and anti-bacterial properties.

Peptide	Sequence	Properties	References
Piscidin 1	FFHHIFRGIVHVGKTIHRLVTG	Anti-viral, anti-bacterial, anti-fungal, anti-parasitic and anticancer	[44,57]
Piscidin 2	FFHHIFRGIVHVGKTIHKLVTG-NH2	Anti-viral, anti-bacterial, anti-fungal, anti-mold and anti-parasitic	[48,58,59,60,61]
Piscidin 3	FIHHIFRGIVHAGRSIGRFLTG	Anti-viral, anti-bacterial, anti- fungal and antiparasitic	[42,58,59,60]
Piscidin 4	FFRHLFRGAKAIFRGARQGXRAHKVVSRYRNRDVPETDNNQEEP	Lowest hemolysis	[61,62]
Piscidin 5	LIGSLFRGAKAIFRGARQGWRSHKA	Anti-bacterial, anti-fungal and anti-parasitic	[63,64]
Piscidin 6	N/A	Antibacterial	[42]
Piscidin 7	N/A	Antibacterial	[42]

**Table 2:** List of pathogens influenced the up and downregulation of piscidin protein genes and organisms.

Pathogens	Upregulation/ Downregulation	Organism	References
Gram-negative bacteria			
*Aeromonas salmonicida*	Upregulation	*Gadus morhua*	[65]
*Edwardsiella tarda*	Up and downregulation	*Oplegnathus* *fasciatus*	[66]
LPS	Upregulation	*Siniperca chuatsi, Chionodraco hamatus*, *Epinephelus coioides*	[67,68,69]
*Vibrio anguillarum*	Up and downregulation	*Dicentrarchus* *labrax*	[42]
Gram-positive bacteria			
*Streptococcus iniae*	Upregulation	*Oplegnathus* *fasciatus*	[66]
Virus and viral analogue			
*Lymphocystis iridovirus*	Upregulation	*Sparus aurata*	[70]
Poly I:C	Upregulation	*Chionodraco hamatus*, *Epinephelus coioides*	[67,68]
Red seabream iridovirus (RSIV)	Upregulation	*Oplegnathus* *fasciatus*	[66]
Parasites			
*Acanthocephalus lucii*	Upregulation	*Perca fuviatilis*	[71]
*Chondracanthus, goldsmid*	Upregulation	*Latris lineata*	[72]
*Cryptocaryon irritans*	Upregulation	*Pseudosciaena* *crocea*	[73]
*Ergasilus sp.*	Upregulation	*Sparus aurata*	[74]

## Data Availability

The data presented in this study are openly available in: Fernandes et al. 2010 https://doi.org/10.1371/journal.pone.0009501; The data presented in this study are available on request from the corresponding author: Capillo et al. 2021 https://doi.org/10.1016/j.fsi.2021.02.006. Rakers et al. *J. Invest. Dermatol.* **2013**, *133*, 1140–1149 https://doi.org/10.1038/jid.2012.503. Zaccone et al. 2006 https://doi.org/10.1002/jez.a.267. Zaccone et al. 1992 https://doi.org/10.1111/j.1463-6395.1992.tb01185.x.
